# The dual nature of partisan prejudice: Morality and identity in a multiparty system

**DOI:** 10.1371/journal.pone.0219509

**Published:** 2019-07-16

**Authors:** Hugo Viciana, Ivar R. Hannikainen, Antonio Gaitán Torres

**Affiliations:** 1 Juan de la Cierva Research Fellow, Universidad de Málaga, Departamento de Filosofía, Málaga, Spain; 2 Institute for Advance Social Studies (IESA-CSIC), Córdoba, Spain; 3 Department of Law, Pontifical Catholic University of Rio de Janeiro, Rio de Janeiro, Brazil; 4 Universidad Carlos III, Departamento de Humanidades, Filosofía, Lenguaje y Literatura, Madrid, Spain; Saint Peter's University, UNITED STATES

## Abstract

Rising hostility between members of opposing political factions has gained considerable attention in both academic and popular press. The adverse effects of this phenomenon are widely recognized, but its psychological antecedents remain the focus of ongoing debate in political psychology. Past research has honed in on two conflicting explanations: one highlights the extent to which people self-define as supporters of particular parties or candidates (the *identity* view), and another points toward the intensity with which they disagree on substantive matters of policy (the *issues* view). A nationally representative survey of 1051 eligible Spanish voters yielded support for both explanations. The perceived magnitude and nature of disagreement were associated with increased partisan prejudice, while controlling for partisan identification. Path analyses revealed that issue-based prejudice was more pronounced among ideologically extreme agents (*β* = 0.237, 95% CI [0.174, 0.300]) than toward extreme targets (*β* = 0.140, 95% CI [0.078, 0.201]), and replicated recent findings that identity-based prejudice is motivated primarily by non-instrumental factors (*β* = 0.286, 95% CI [0.230, 0.337]). Together, these results indicate that discrimination across party lines responds to two fundamentally distinct, though at times co-occurring, imperatives: to coalesce in ideologically homogeneous communities, and to protect one’s sense of partisan identity.

## Introduction

Recent years have witnessed a surge of interest in the study of partisan prejudice. Whereas explicit racial and sexual discrimination are strongly policed and may even be in decline, discrimination against members of ideological outgroups is not clearly sanctioned and has been identified from many disciplinary perspectives as a rising form of prejudice [1], [2].

Laboratory studies demonstrate that supporters of ideologically-opposing political parties elicit prejudice, as assessed through both self-report and behavior [3]. Corresponding effects of partisanship on interpersonal trust have been documented throughout nations with varying degrees of political diversity and social cohesion [4]. Importantly, partisan prejudice may have profound consequences outside the lab, for instance, by contributing toward geographical assortment [5] and a net reduction in social trust [6], [7], [8], [9].

Existing work describing the origins of prejudice toward opposing political parties has supplied two distinct explanations. One of these is centered on the importance of partisan attachment and *identity* [10], [11] [12], [13], [14], [15], while the other focuses on the role of prescriptive disagreement in ethical and policy *issues* [16], [17], [18], [19], [20], [21], [22], [23]). We summarize these distinct bodies of evidence in Section 1.1, before turning to the main innovations of our approach.

First, despite robust evidence that moral disagreement underlies partisan prejudice [24], [25] no previous studies have specifically investigated whether *meta-ethical* beliefs about the nature of such disagreement predict prejudice in the political context. In Section 1.2, we draw on the distinction between (first-order) moral values and (second-order) meta-ethical beliefs to motivate our hypothesis that these constructs independently predict prejudice.

Second, several studies have documented ideological and affective polarization in the United States, such that Republicans and Democrats alike are more extreme in their moral views [3], [1] and also more prejudiced against each other [26]. By examining polarization in the context of multiparty politics, we assess whether prejudicial attitudes are more pronounced among extreme agents or toward extreme targets, a distinction that has thus far received limited attention and yielded inconclusive answers, as detailed in Section 1.3.

### Issue-based and identity-based theories of partisan prejudice

Although partisan prejudice is now widely documented, its underlying causes are not yet well understood. One series of studies has concluded that partisan prejudice may reflect concrete ideological disagreement [18], [27], [28], *i*.*e*., a preference for (or prejudice against) social partners to the extent to which prescriptive agreement (or, respectively, disagreement) is perceived [24], [25]). In other words, the belief that rival-party supporters disagree with us on important issues—whether these be economic policy, same-sex rights, or immigration—triggers our prejudice against them [17], [19], [20]. If those issues are perceived as moral, the reverberations might be even louder.

Though the extent of such disagreement is often exaggerated [29], [30] mounting evidence demonstrates that the moralities of liberals and conservatives reliably differ [31], [32], [33], [34]. Furthermore, numerous policy issues are framed in moral language [35], [36], [37], [38], and their moralization leads to increased social distance [25] and stigmatization [36] and can also spark violent behavior [21]. Since moral values often shape policy proposals and legislation, strategically ostracizing discordant moral voices may help to advance one’s preferred policies [24]. In line with this, moral disapproval of opposing party leaders predicts increases in hostility over time [16]. The effect of moral disagreement on prejudicial attitudes has also been observed in an experimental context [23], by randomly assigning participants to report their feelings toward ideologically moderate versus extreme members of their opposing party.

Building on a long tradition of uncovering ingroup bias across a vast array of demographic traits (e.g., ethnicity, nationality, and religion), some fascinating research points toward a different source of partisan prejudice. In particular, a growing literature under the banner of *expressive partisanship* has now shown that rudimentary feelings of partisan attachment and *identity* also fuel conflict between partisans across the left-right divide [12], [39], [40], [41]. A sense of partisan attachment appears to mobilize voters to a greater extent than ideological syntony, as documented by studies of voting behavior and campaign involvement [10], [12]. Stronger partisan identities also magnify feelings of anger toward rival-party supporters [15] at the prospect of electoral loss, while predicting enthusiasm and overconfidence in electoral victory [11], [12]. Collectively, these studies open up the possibility that growing partisan prejudice is driven not by real or perceived division on concrete policy questions, but by primitive instincts propelling identity-based opposition.

Some work has argued that this distinction between issue-based and identity-based prejudice is fundamentally cosmetic, and that feelings of partisanship serve an *instrumental* purpose. In other words, to the extent that citizens are opinionated on current ethical and policy issues, they will acquire partisan identities with which to symbolize their ideological commitments [42], [43], [44], [45]. These partisan identities will then appear to predict their hostility toward rival party members, but this hostility is ultimately a reflection of substantive ideological disagreement and a means of collectively advancing shared policy preferences in the public forum [46], [47].

### Deepening the issues view: Do meta-ethical beliefs play a role?

Not all disagreements spark prejudice to the same degree [48]. To illustrate this, imagine you face two contenders in disagreement: The first disagrees with you on the famously divisive culinary question of whether cilantro is foul or fragrant and delicious. The second believes that God created the Earth in six days. You may find yourself more willing to befriend the former than the latter, and research comparing the interpersonal consequences of disagreement attests to this general trend [19], [27].

It stands to reason that, if we think of disagreement on prescriptive questions more like the expression of differing taste or personal preference, we may have little reason to discriminate against people who disagree with us. If, however, we think of prescriptive disagreement as factual contradiction, then we will likely view our contenders in debates about morality and policy like we see creationists or flat-earthers: as mistaken in fundamental ways.

Among philosophers, beliefs about the nature of normative disagreements are known as *meta-ethical* beliefs [49], [50]. Meta-ethical beliefs capture the status or grounding of our moral opinions, i.e. whether they refer to an objective reality or result from local customs or conventions. In philosophical parlance, we make meta-ethical claims when we say things like ‘it is *a fact* that torturing babies for fun is wrong’ or ‘murder is wrong *here and everywhere*’. We also make meta-ethical claims when we describe other second-order properties of our moral views, such as their *scope* (whether they apply locally or universally), their *centrality* for our life [51] or even the *importance* of moral obligations when compared to other systems of norms (law, etiquette, etc.) Normative beliefs or first-order moral judgments, in contrast, are expressed through unqualified assertions such as ‘torturing babies is wrong’ or ‘murder is wrong’.

To date, a handful of studies have outlined the contours of meta-ethical belief in the general public [52], [53], [54], [55]. These studies typically expose participants to specific prescriptive disagreements, and ask a diagnostic question: Can only one contender be right and thus the other must be wrong, or, alternatively, could they both be right? When thinking about divisive issues such as abortion, stem cell research or assisted suicide, people sometimes adopt a more *relativistic* stance and believe that both could be right ([56], [57]). In contrast, highly consensual moral norms are seen as more *objective* ([58], [57]), rendering the belief that, for example, cheating or theft is OK, objectively false in most people’s eyes. With age, the belief that morality is objective tends to crystallize [59], but people can be nudged toward a relativistic view by thinking about morality in a distant culture [55] or by framing ethical debate as a cooperative learning opportunity [60].

In the previous exercise, we suggested that acquaintances with divergent culinary preferences are more likeable than those with whom we disagree about basic matters of fact. Evidence from experimental ethics suggests that whether we view our moral disagreements more like the former or the latter also predicts how tolerant we will be of each other [61]. Specifically, the belief in an objective moral truth is linked to greater prejudice, while viewing moral disagreement as the expression of subjective preferences is linked to greater tolerance [56]. This effect has been shown not only in peripheral issues, but also in issues that we care deeply about [62], indicating that meta-ethical beliefs are dissociable from the strength of one’s personal conviction.

Despite growing evidence that relativist meta-ethical beliefs predict tolerance toward others [63], and a wide recognition of the role that morality plays in processes of polarization [24], [25], to the best of our knowledge no previous work has specifically linked this construct to the study of partisan prejudice. Researchers have debated whether prejudice results from disagreement on issues or identity. Many have found that disagreement on issues plays a weak role, and argued that partisan identity is the primary motivator. It could be speculated, however, that the reason that these researchers may have found weak effects of issues is that they assumed that prejudice would arise merely as a result of *first-order* disagreement on issues. But as we noticed above, there is evidence that prejudice is exarcebated when disagreements are seen as objective. This brings us to our first research question:

**Question 1.** Do the *extent* of moral disagreement and beliefs about the *nature* of moral disagreement predict independent variance in partisan prejudice?

Question 1 leaves an interesting path to explore. Meta-ethical beliefs are about moral opinions, so an obvious thing to ask is if meta-ethical beliefs can interact with the way we perceive others’ moral opinions in cases of disagreement, modulating our reactions to them. One intuitive characterization of the role of meta-ethics suggests that relativists should see different moral communities as equally entitled to their values [49], whereas objectivists—by virtue of the belief that there is a single moral truth—should prefer to interact with the morally congenial, and eschew contact with those perceived as manifestly wrong about how things *should* be. This interpretation rests on a prediction that we are in a position to test: namely, that as perceived disagreement *decreases*, so should the difference between objectivists’ prejudice and relativists’ tolerance (see Hypothesis 3).

### Ingroup homophily versus outgroup derogation

In the empirical literature documenting the effects of ideological disagreement on prejudice, disagreement is often instrumentalized as the brute ideological distance between two parties. It is then observed that this absolute difference (i.e. the extent of disagreement) is associated with an outcome measure of prejudice (e.g., *feeling thermometers* see [23] or *likes/dislikes* in [26]). Collectively, these studies have yielded an understanding of polarization as mutual and undifferentiated disdain between partisans on the left and right.

**Fig 1:**
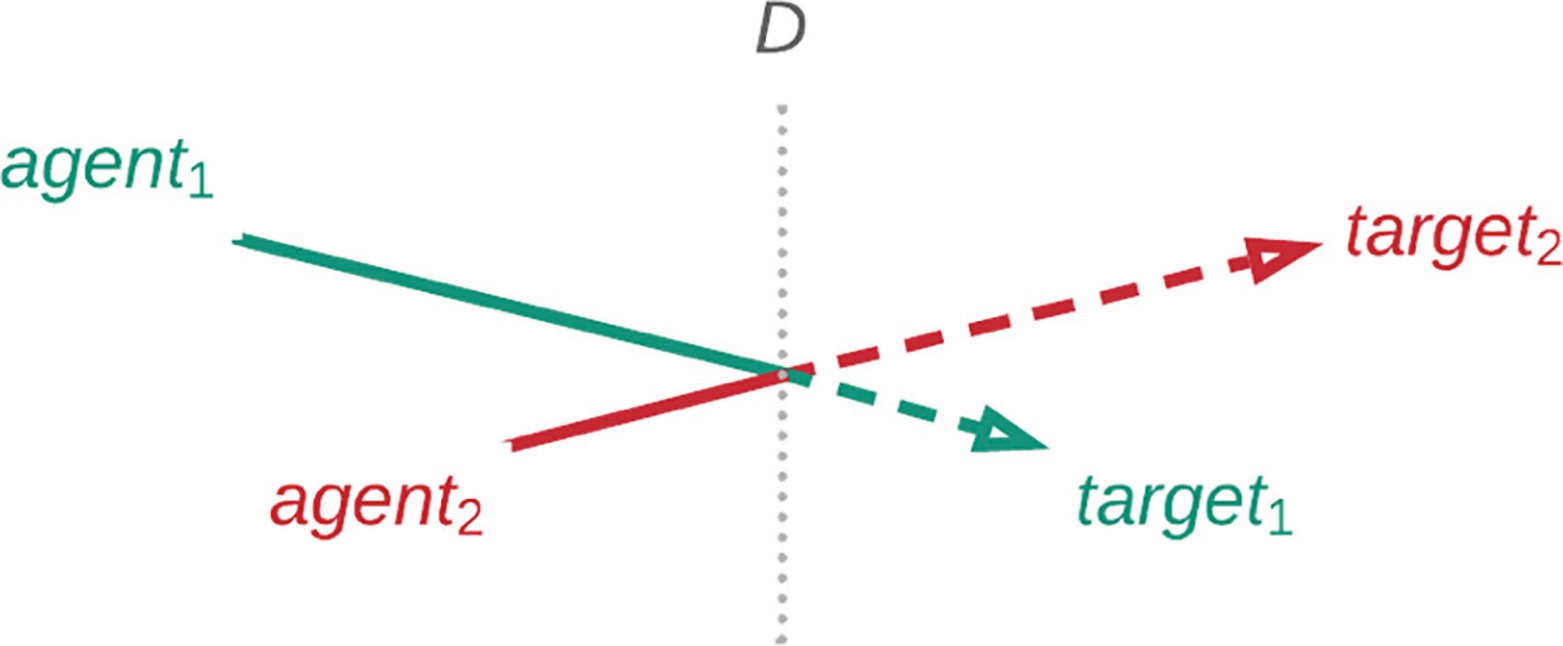
Schematic diagram of ideological disagreement. Segmenting disagreement at the left-right divide reveals two distinct components: agent extremity (solid section) and target extremity (dashed section).

A second contribution of our present study is to sharpen this somewhat coarse-grained picture of partisan prejudice by defining two conceptually distinct forces potentially at play. As depicted in Fig 1, most ideological disagreements occur between citizens on opposing sides of a left-right divide. By reference to this divide, we can think of ideological disagreement as composed of agent extremity plus target extremity, which leads to the second question guiding the design of our study:

**Question 2.** Is partisan prejudice greater among more extreme agents, or toward more extreme targets?

Partisan prejudice could arise as a function of overall disagreement and, therefore, depend equally on agent and target extremity. Yet this need not be the case: Prejudice could depend to a greater extent on agent extremity (in which case Agent 1 should reveal greater prejudice), or target extremity (in which case Target 2 should be subject to greater prejudice).

This distinction may not only yield insight into the mechanism of partisan prejudice, it could also hint toward its ultimate *purpose*: If prejudice is greater toward extreme than moderate targets, this would suggest that partisan prejudice contributes to the regulation of existing moral communities, i.e. by ostracizing and derogating nonconformists. If prejudice is greater among ideologically extreme than moderate agents, this may indicate that partisan prejudice plays a role in the establishment of new moral communities through homophily among extreme ideologues.

Research on affective polarization has largely overlooked the possibility of distinguishing between these hypotheses. This is understandable since, though conceptually distinct, agent and target extremity are somewhat difficult to dissociate empirically. A vast majority of the research on partisan prejudice—and political polarization more broadly—has focused on polarization in the United States electorate. In a two-party system especially, as is the case in the US, agent and target moral values are likely to be strongly opposed, imposing a certain degree of negative collinearity and thereby limiting analysts’ capacity to statistically dissociate their effects.

To get around this issue, we conducted our study in an ideologically diverse electoral context, namely, Spain. In Spain, there are four primary parties and, in recent elections, each has secured a considerable fraction of the popular vote. *Partido Popular* (PP, “People’s Party”, http://www.pp.es) and *Partido Socialista Obrero Español* (PSOE, “Spanish Socialist Workers’ Party”, http://www.psoe.es) have been the conservative and progressive establishment parties respectively, roughly since the transition to democracy (1975–1982). Both parties are strongly pro-European, and have alternated in government since 1982 (PSOE from 1982 to 1996, and from 2004 to 2011; PP from 1996 to 2004 and from 2011 to 2018). In the 2016 general election, PSOE received 22% and PP received 33% of the vote. Much of the remaining vote was captured by two relatively new parties: *Ciudadanos* (Cs, “Citizens”, https://www.ciudadanos-cs.org/) received 13%, and *Unidos Podemos* (UP, “United We Can”, https://podemos.info/) received 21%. Cs and UP consolidated in relatively recent years, leveraging resentment toward establishment politics and attracting the youth vote. Cs is a center-right pro-European party, founded in 2008 in Catalonia. UP is a left coalition that grew out of *Podemos*, a party founded in 2014 that advocates wide progressive reform in the wake of the economic recession.

Multi-party systems have been shown to emerge when the electorate holds more diverse moral and social views (Ezrow, 2007), and this seems true of Spain, where two parties cater to hardline progressive (Unidos Podemos) and conservative (PP) voters, and two parties cater to moderate voters on the center-left (PSOE) and center-right (Ciudadanos). Though we expect to see substantial variation in voters’ own values, since supporters of moderate parties are foreseeably less likely to be the targets of prejudice, the variance in rival ideologues’ perceived values should remain limited in comparison (an asymmetry we indeed find at the party level; see section ‘Descriptive statistics’). In these conditions, agent and target extremity should be *less* correlated overall, which in turn enables us to detect any differences in their linear contribution to issue-based prejudice.

Finally, in order for our conclusions to be externally valid, we required an accurate estimate of the population’s left-right divide. Thus, we turned to representative sampling methods and derived a simple numerical optimization algorithm to locate the left-right divide.

Juxtaposing Questions 1 and 2 forms the basis of our study protocol (see Table 1). Each participant is asked about their own moral values and meta-ethical beliefs, and also asked to imagine the moral values and meta-ethical beliefs of a third-party target.

**Table 1:** A compositional perspective on moral disagreement.

Example issues	“Abortion is morally wrong” “Environmental standards should be flexible given the importance of economic growth” “Spaniards should be loyal to their country above other concerns”	
	Moral views (-3: “*Strongly disagree*”– 3: “*Strongly agree*”)	Meta-ethical beliefs (1: “*Only one can be right*”, 0: “*Both can be right*”)
Self-report	According to your values and personal opinions, to what extent do you agree or disagree with each of the following statements?	Imagine that two people are arguing about whether the statement is true or false. One person thinks it is true and the other thinks it is false. In your opinion, can both be right or is one of them mistaken?
Target representation	Think about people who, from among the four main political parties, are most in favor of [least-liked party]. In this section, we want to know whether you can guess what people who support [least-liked party] tend to believe. To what extent do people who support [least-liked party] agree or disagree with each of the following statements?	Think about people who, from among the four main political parties, are most in favor of [least-liked party]. In this section, we want to know whether you can guess what people who support [least-liked party] tend to believe. Imagine that two people are arguing about whether the statement is true or false. One person thinks it is true and the other thinks it is false. For someone who supports [least-liked party], can both be right or is one of them mistaken?

## Methods

The present study was funded by the Ministerio de Economía y Competitividad (Convocatoria 2015 Programa Estatal de Investigación, Desarrollo e Innovación Orientada a los Retos de la Sociedad) and was reviewed and approved by the ethical board ‘Comité de Ética del Consejo Superior de Investigaciones Científicas’. The informed consent sheet was approved by the ethics committee and online written consent was obtained from invited participants before taking part in the survey.

Below we report how our sample size was determined, and describe all planned procedures and measures used in the study. Procedures, experimental predictions, and analysis plans were pre-registered on the *Open Science Framework* at: https://osf.io/kj6ep/.

### Scale development and pilot data

Before launching the national survey, we piloted our assessments of moral values and partisan prejudice.

### Moral values

Since grounds for ideological confrontation vary widely from country to country (e.g., gun laws are a core issue in the United States but not in Spain), and most validated instruments were developed for purposes of research on United States politics, we were forced to develop a novel culture-specific measure.

By taking into account the primary culture-war issues in Spain today, we first drafted eighteen prescriptive statements, some of which capture characteristically conservative views, and others characteristically progressive views (listed in table A S1 Appendix). To examine the reliability and factor structure of our items, we conducted a pilot study, recruiting a convenience sample of 261 Spanish residents (77% women, *M*_age_ = 34 years old) through an ad on Facebook. Pilot respondents were asked whether they themselves (Agent condition, *n* = 137) or supporters of their least-liked party (Target condition, *n* = 124) agree with each statement on a scale from -3: “Strongly Disagree” to 3: “Strongly Agree”.

Since we fell short of the recommended sample size to conduct separate principal components analysis by condition, and our interest is in the factor structure of our items independently of whether participants self-report or describe their ideological rivals, we conducted a principal components analysis on the pooled (Agent and Target) data. Two items failed to load onto the principal factor: *Surrogacy*, and *Prostitution*. This resulted in a sixteen-item index of moral values that demonstrated very good reliability in both the Agent (Cronbach’s *α* = .81) and Target (Cronbach’s *α* = .95) conditions.

### Partisan prejudice

For our assessment of partisan prejudice, we adapted a social distance measure developed by Almond and Verba [64], previously employed in numerous studies on polarization (e.g., [3], [25], [26], [14]). Respondents were asked how much they would like or not like to establish four relationships with an ingroup (i.e. supporter of their preferred party) and an outgroup (i.e. supporters of their disfavored party) member, on an 8-point scale. For our pilot data, every item loaded onto the principal component (see table B in S1 Appendix). Selective social distancing from outgroup members was observed across every item pair, *t*s > 3.90, *p*s < .001, and the eight-item partisan prejudice index revealed good reliability (*α* = .83).

## Sample size calculation

We wanted to reliably detect effects as small as *η*_p_^2^ = .02, or approximately, *r* = .14—a conservative estimate of the effect, also smaller than the average effect in social psychology [65]. Setting both Type-1 and Type-2 error rates to .05, a power analysis for ANOVA recommended a target sample size of 650. In order to adequately represent supporters of each political party, we were advised by the survey company (IMOP) to recruit at least 1000 participants.

## Participants

We partnered with IMOP (https://www.imop.es), which is a leading market research and polling firm based in Madrid, Spain, to obtain a representative sample of the Spanish adult population. The IMOP panel is a probabilistic sample of the Spanish population recruited through random digit dialing. Opt-in participation is not allowed, and the panel is continuously updated.

Between October 23rd and November 13th 2018, a total of 3055 IMOP panelists were invited to take part in our study by visiting a survey website. No more than five reminders were sent to each invitee. At the end of the three-week data collection window, 1051 Spanish adults (552 women, M. age = 43.8) had completed the study, yielding a response rate of 34.4%. See S1 Appendix for a comparison between our survey sample and national census data on basic demographic measures.

Frequency weights (*M* = 0.95, *SD* = 0.25, *Min* = 0.53, *Max* = 2.30) were calculated via inverse probability of selection and applied to the final sample on three demographic parameters: (1) *region* (seven geographical regions), (2) *age-by-sex*, and (3) *settlement size* (five levels, from urban to rural). Three pre-registered exclusion criteria were implemented by the survey company prior to handing over the data (see S1 Appendix for details).

## Procedure

All text and materials for the online survey were administered in Spanish (see S1 Appendix for verbatim stimuli and English translations).

After providing written consent, participants completed four tasks: (1) *Identification*, which was always presented first. Participants were then presented with the (2) *Self* and (3) *Target* blocks in a counterbalanced order across participants. Finally, participants completed the (4) *Partisan prejudice* assessment.

### Identification

Participants were asked to report the extent to which they like and identify with each of the four major political parties in Spain: *Partido Popular* (PP), *Partido Socialista Obrero Español* (PSOE), *Unidos Podemos* (UP), and *Ciudadanos* (Cs). Each participant had to rank order the political parties from 1: ‘most liked party’ to 4: ‘least liked party’. In addition, participants rated the degree to which they identify with each political party, on a scale from 0 (‘Not at all’) to 100 (‘Absolutely’).

### Self

For each of the sixteen divisive moral statements (e.g., “The voluntary termination of pregnancy is immoral”), participants were asked two questions to independently assess their moral values and meta-ethical beliefs, as shown in Table 1.

**Fig 2:**
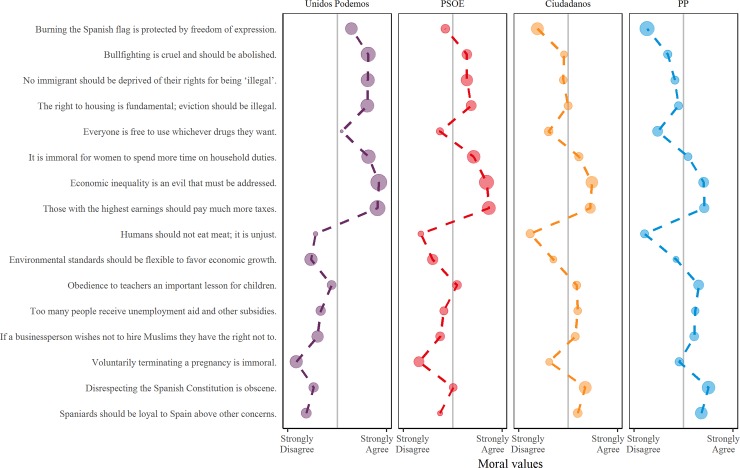
Agreement with each statement by preferred party. Large points represent objective issues, while small points represent relativist issues.

To capture participants’ moral values, we asked them whether they agree or disagree with each statement on a scale from -3: “Completely disagree”, to 3: “Completely agree”. Then, to assess their meta-ethical beliefs (about whether there is an objectively right view on each issue), we adapted a popular paradigm in moral psychology (see e.g., [52], [59], [53], [66], [67], [68]). Specifically, participants were asked to imagine that two people disagree about whether the statement is true or false, and then report whether 0: “Both people could be right” or 1: “Only/at most one person could be right” (with the alternative to opt out, i.e. answer “I don’t know/I don’t want to answer”). Data for the Self block by party preference are displayed in Fig 2.

### Target

Participants were also asked to consider their disfavored political party (ranked last in the initial *Identification* task), and answer all sixteen moral values and meta-ethical belief questions as if they were a typical supporter of their disfavored political party.

All divisive moral statements along with filler statements about matters of fact (e.g.: “The Earth is flat and the sun rotates around the Earth”), taste (e.g., “Tea tastes better than coffee”), and one apolitical moral statement (“It is wrong to cause unjustified and gratuitous suffering to innocent people”) were presented in a random order.

### Partisan prejudice

For both supporters of their preferred (1^st^) and disfavored (4^th^) political parties, each participant was asked “How much would you like for your”:

1. “Brother/sister or daughter/son to marry a supporter of” [preferred/disfavored party],
2. “Boss to be a supporter of” [preferred/disfavored party],
3. “Children’s school teacher to be a supporter of” [preferred/disfavored party], and
4. “Neighbor to be a supporter of” [preferred/disfavored party].

Each answer was recorded on a scale from 1: “I would very much dislike it” to 8: “I would very much like it”. The average difference (preferred–disfavored) in social distance across all four-item pairs constituted our measure of partisan prejudice.

## Data reduction

Our dependent measure in the analyses below is *partisan prejudice*.

1. **Partisan prejudice:** the mean difference (ingroup—outgroup) across the four item pairs in the social distance task (Cronbach’s *α* = .85), from -7: maximum prejudice toward the ingroup, through 0: no prejudice, to 7: maximum prejudice toward the outgroup.
  Our primary independent measures are *moral disagreement*, *agent* and *target objectivism* and *partisan identification*.
2. **Moral disagreement:** the mean absolute difference between *agent values* (Cronbach’s *α* = .84) and *target values* (Cronbach’s *α* = .95) on all sixteen moral value items, ranging from 0: no disagreement to 6: maximum disagreement.
  Moraldisagreement=|agentmoralvalues‐targetmoralvalues|
3. **Agent and target objectivism**: the proportion of objectivist meta-ethical beliefs (Cronbach’s *α* = .75, .89) across all sixteen items in the *Self/Target* block, from 0: *non-objectivist* to 1: *objectivist*.
4. **Partisan identification**: the difference in identification (Cronbach’s *α* = .82) with the preferred party versus the least-liked party, from -100 (total identification with the disfavored party) through 0 (no relative identification) to 100 (total identification with the preferred party).

**Fig 3:**
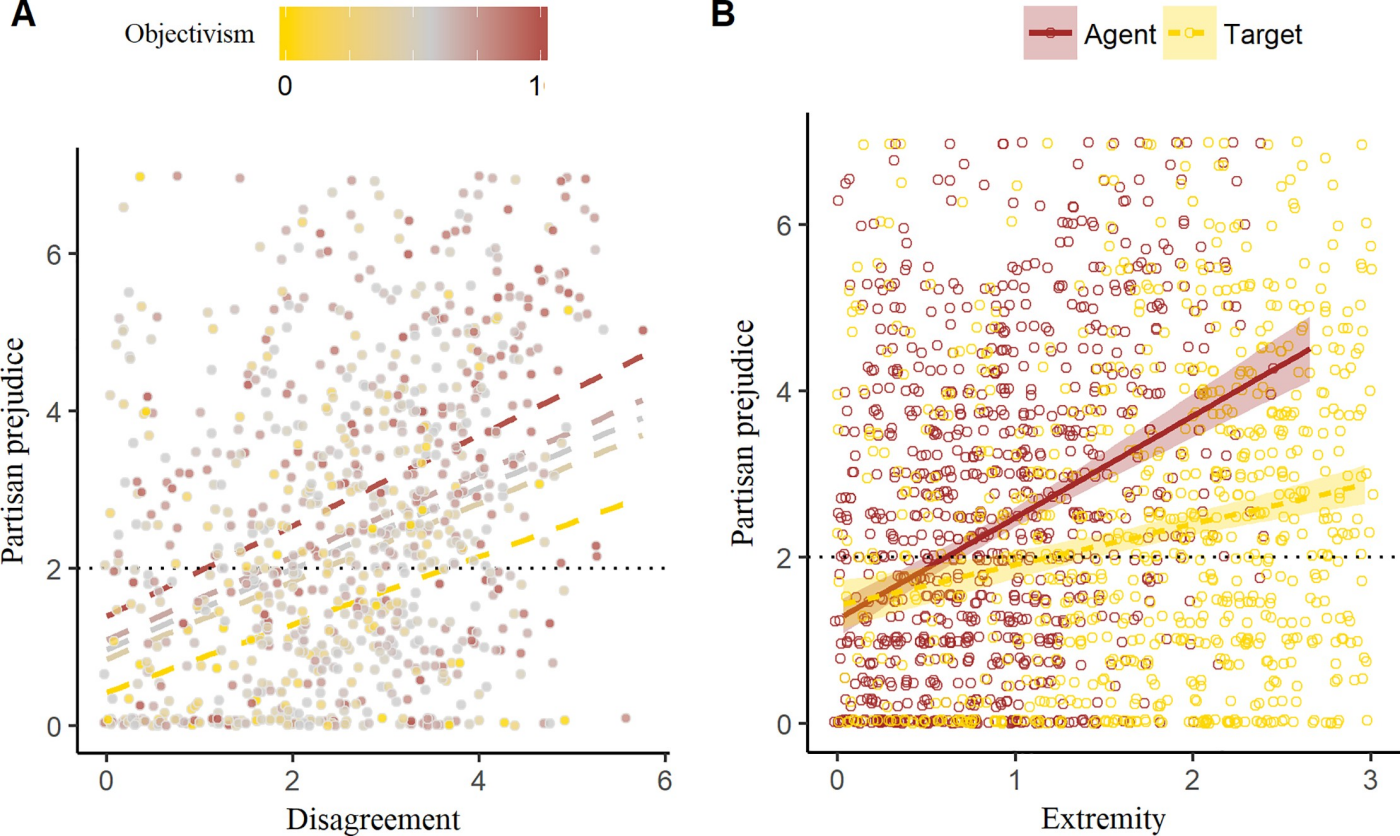
Observed values of partisan prejudice by (A) moral disagreement and (B) extremity. (A) Linear trend lines show the predicted prejudice at different values of meta-ethical belief: Minimum (yellow), Q_1_, Median, Q_3_, and Maximum (brown). The separation between the lines reveals the main effect of meta-ethical belief, while their similar slope attests to the absence of an interaction with disagreement. (B) Linear trend lines show the predicted values of prejudice as a function of agent and target extremity. Both y-axes have been truncated at 0.

In the exploratory results section (3.3.1), we describe a procedure for estimating the left-right divide, *D*. Having calculated the position of the left-right divide, two further variables were then computed:

5 **Agent and target extremity**: the absolute difference between moral values and the left-right divide, *D* (see Fig 3B), in the *Self/Target* blocks:
  Agentextremity=|agentvalues‐D|
  Targetextremity=|targetvalues‐D|

Higher values indicate more extreme views—whether progressive or conservative—and lower values indicate more moderate views.

## Predictions

We pre-registered three predictions regarding the relationship between moral disagreement, meta-ethics and partisan prejudice:

**Hypothesis 1.** Moral disagreement predicts partisan prejudice (controlling for objectivism).
**Hypothesis 2.** Objectivist beliefs predict partisan prejudice (controlling for moral disagreement).
**Hypothesis 3.** Objectivist beliefs moderate the effect of moral disagreement: i.e. moral disagreement better predicts partisan prejudice among objectivists than among relativists.

Thus, while Hypotheses 1 and 2 predict omnibus effects of disagreement and objectivism respectively, Hypothesis 3 predicts an interaction between moral disagreement and objectivist beliefs about the nature of moral disagreement. We then followed up on any of the Hypotheses 1–3 that were borne out, by examining whether these relationships are driven by self-reported morality (objectivism and/or extremity), representations of target morality, or both.

## Analysis plan

We first model *a*’s prejudice toward *t*, *P*_*at*_, as a function of five morality predictors: moral disagreement between *a* and *t* (*D*_*at*_), agent (*O*_*a*_) and target (*O*_*t*_) objectivism, and the two-way interactions between disagreement and each measure of objectivism:

$$P_{at}=\alpha +\beta_{1}D_{at}+\beta_{2}O_{a}+\beta_{3}O_{t}+\beta_{4}DO_{a}+\beta_{5}DO_{t}+\varepsilon \;[\mathrm{morality}‐\mathrm{only}]$$

We refer to this regression model as the *morality-only* model, and conduct model comparison tests for three models nested within it. To investigate the effects of disagreement (Hypothesis 1) and objectivism (Hypothesis 2), we compare the morality-only model to a model without the corresponding terms or their higher-order interactions. Then, to investigate the prediction that objectivism moderates the effect of moral disagreement (Hypothesis 3) we drop only the two-way interactions. For each hypothesis test, we report the change in variance explained, Δ*r*^2^, as the corresponding measure of effect size.

To evaluate whether disagreement and objectivism remain significant when controlling for identity, we enter the identification term, *Id*, in the *morality-plus-identity* model:

$$P_{at}=\alpha +\beta_{1}D_{at}+\beta_{2}O_{a}+\beta_{3}O_{t}+\beta_{4}DO_{a}+\beta_{5}DO_{t}+\beta_{6}Id+\varepsilon \;[\mathrm{morality}‐\mathrm{plus}‐\mathrm{identity}]$$

We report the standardized effect size (with *M* = 0 and *SD* = 1) of every predictor to render the coefficients of our regression analyses interpretable and comparable.

To answer Question 2, we compare the contributions of agent and target extremity to partisan prejudice by replacing the disagreement term with both extremity terms, *E*_a_ and *E*_t_. In Exploratory Analysis 1, we compare their magnitude via linear hypothesis tests [69].

In Exploratory Analysis 2, we model partisan identification in terms of extremity and objectivism:

Id=α+β1Ea+β2Et+β3Oa+β4Ot+β5DOa+β6DOt+ε

and report their direct and indirect effects on partisan prejudice.

In S2 Appendix, we report further exploratory tests which, though tangential to the primary objectives of our report, yield insight into the generalizability of past findings regarding the *misrepresentation hypothesis* ([30], [70], [71]; see S2 Appendix section 2 and 3) and the *ideological symmetry vs*. *asymmetry debate* ([72], [73]; see S2 Appendix section 4).

## Results

Table 2 displays an overview of key results, separating confirmatory analyses, H_1_- H_3_, from exploratory and supplementary analyses. For each confirmatory analysis, we also report whether the results we obtained conform to our pre-registered predictions, accompanied by the corresponding Bayes Factor in favor of the alternate hypothesis.

**Table 2:** Overview of key results.

Section		Main results	As predicted	BF_10_
3.2	H1	Moral disagreement predicted partisan prejudice.	Yes	4 × 10^26^
	H2	Objectivist beliefs predicted partisan prejudice.	Yes	19.1
	H3	Objectivist beliefs did not moderate the effect of moral disagreement on partisan prejudice.	No	0.03
3.3	X1	Prejudice was greater among ideologically extreme agents than toward ideologically extreme targets.	-	
	X2	Extremity and identity have largely independent effects on partisan prejudice.	-	
Appendix	S2	Participants exaggerated the extremity of ideological targets.	-	
	S2	We observed left-right symmetry in processes of partisan prejudice.	-	

### Descriptive statistics

Table 3 displays descriptive statistics broken down by party preference. We observed the expected linear trend in values across parties, *z* = -23.3, *p* < .001, and greater by-party differences in values for agent than target reports, indicating that by sampling from a multiparty electoral system we succeeded in decorrelating agent and target extremity for purposes of addressing Question 2. This agent-target discrepancy is consistent with the predicted under-representation of moderate parties as targets, i.e., a McNemar-Bowker Test revealed dependence between parties and ranks, χ^2^(df = 6) = 256.9, *p* < .001, but also reflects exaggerated disagreement with hardline party supporters (discussed in S2 Appendix). By-party differences in partisan prejudice were also observed, with greater prejudice among hardline parties than moderate parties.

**Table 3:** Descriptive statistics including by-party preference.

		By party preference			
	Total	Unidos Podemos	PSOE	Ciudadanos	Partido Popular
*Descriptors*		*Newly-formed*	*Established*	*Newly-formed*	*Established*
		*Hardline*	*Moderate*	*Moderate*	*Hardline*
		*Progressive*	*Progressive*	*Conservative*	*Conservative*
*Age (years)*	44.4	43.0	45.2	43.3	47.4
*Gender* *(% women)*	52%	54%	53%	50%	52%
*Church attendance* ^*1*^	.19 [.18, .21]	.08 [.06, .10]	.17 [.14, .20]	.24 [.20, .27]	.41 [.35, .47]
*Household income* ^*1*^	.68 [.67, .69]	.69 [.67, .72]	.67 [.65, .70]	.67 [.65, .69]	.67 [.63, .70]
*Educational attainment*^*1*^	.51 [.50, .53]	.51 [.49, .54]	.52 [.49, .55]	.50 [.47, .53]	.52 [.48, .56]
*Partisan identification* ^*1*^	66.6 [64.9, 68.3]	67.2 [64.0, 70.3]	63.9 [60.3, 67.3]	67.6 [64.4, 70.8]	68.6 [64.0, 73.3]
*Target (mode)*	Unidos Podemos (39%)	Partido Popular (55%)	Partido Popular (64%)	Unidos Podemos (83%)	Unidos Podemos (92%)
*Values*	0.51 [0.44, 0.57]	1.43 [1.35, 1.50]	0.69 [0.61, 0.78]	-0.20 [-0.28, 0.11]	-0.59 [-0.70, -0.47]
*Target values*	-0.36 [-0.47, -0.25]	-1.75 [-1.85, -1.64]	-1.12 [-1.29, -0.94]	1.15 [0.98, 1.33]	1.36 [1.17, 1.54]
*Objectivism* ^*1*^	.55 [.54, .57]	.61 [.59, .64]	.53 [.51, .56]	.51 [.49, .54]	.53 [.50, .57]
*Target objectivism* ^*1*^	.71 [.70, .73]	.75 [.72, .77]	.73 [.70, .76]	.69 [.66, .72]	.66 [.62, .71]
*Partisan prejudice*^*1*^	.32 [.30, .34]	.42 [.38, .45]	.25 [.22, .28]	.27 [.23, .30]	.34 [.29, .39]

^1^ = min-max normalized.

Table 4 displays the pairwise correlations between measures of identification, disagreement, meta-ethical beliefs and partisan prejudice, collapsed across party preferences.

**Table 4:** Correlation table of partisan identification, morality measures, and partisan hostility (Spearman’s ρ coefficient, and p value). To eliminate common method variance and estimate the unique relationship between each pair of constructs, we report partial correlations above the diagonal.

		(1)	(2)	(3)	(4)	(5)
(1)	*Partisan identification*	-	.038^#^ .232	.020^#^ .529	.017^#^ .593	.351 .000
(2)	*Disagreement*	.192 .000	-	.120 .000	.280 .000	.292 .000
(3)	*Objectivism*	.112 .000	.223 .000	-	.099 .002	.129 .000
(4)	*Target objectivism*	.094 .004	.332 .000	.170 .000	-	.018^#^ .571
(5)	*Partisan prejudice*	.397 .000	.380 .000	.218 .000	.162 .000	-

Note:

^#^ indicates non-significance (p > .05).

### Pre-registered hypotheses

The morality-only model of partisan prejudice was highly significant, *F*(5, 1045) = 40.76 .13, *p* < .001, and explained 16% of the variance in partisan prejudice. By reference to the morality-only model (*AIC* = 2842), dropping the disagreement (*AIC* = 2961) and objectivism (AIC = 2855) terms reduced model fit: disagreement *F*(1, 1049) = 179.76, Δ*r*^2^ = .08; *objectivism F*(2, 1048) = 32.20, Δ*r*^2^ = .01, both *p*s < .001. These results therefore replicate prior findings and show that moral disagreement (Hypothesis 1) and objectivist meta-ethical beliefs (Hypothesis 2) underlie partisan prejudice.

Hypothesis 3 was not supported: Dropping the interactive terms alone (*AIC* = 2860) did not reduce model fit, *F*(2, 1045) = 0.57, Δ*r*^2^ = .001, *p* = .57. A Bayesian analysis revealed that the additive model provided better fit to the observed data than the interactive model, BF_01_ = 45.9, which constitutes strong evidence that the effect of moral disagreement on prejudice is not moderated by meta-ethical beliefs.

As shown in Table 4, partisan identification also strongly predicted prejudice, conceptually replicating the results of previous research [26], [14]. Compared to the morality-only model (*AIC* = 2842, the morality-plus-identity model of partisan prejudice (AIC = 2720) substantially improved model fit, *F*(1, 1044) = 99.96, Δ*r*^2^ = .10, *p* < .001, and explained 26% of the variance in partisan prejudice.

The effects of disagreement (*β* = 0.28, *t* = 9.97) and identification (*β* = 0.31 *t* = 11.45) were roughly comparable on a standardized scale, while the effect of objectivism was somewhat smaller, (*β* = 0.10, *t* = 3.55), all *p*s < .001. Nonetheless, all three effects were highly significant, and the remaining terms revealed no independent effects, | *β* |s < 0.03, |*t|*s < 1, ps > .25.

A comparison of the relative importance of predictors in the morality-plus-identity model, using the R package ‘relaimpo’ [74] revealed that partisan identification predicted 12% of the variance, whereas moral disagreement predicted 10% of the variance in partisan hostility (as calculated by the metric lmg, *R*2 partitioned by averaging over orders, as recommended in [75]. Agent objectivism predicted an extra 2.5% of the variance by the same metric.

#### Exploratory analyses

A second objective of our report was to distinguish agent extremity from target extremity (which we define as the absolute distance from the ideological center) and to assess whether they differentially predict partisan prejudice (as depicted in Fig 1).

#### Finding the left-right divide

We cannot simply define the ideological center as either the arithmetic mean of moral values (since this estimate would be too sensitive to the presence of outliers, such as extremist groups) or the median (since this would assume that there are equal numbers of progressives and conservatives).

Instead, we define the left-right divide by reference to one of its more indisputable features: i.e. progressives are on its left and conservatives on its right. Thus, our task was to find the point along the ideological spectrum that best distinguishes progressives from conservatives.

**Fig 4:**
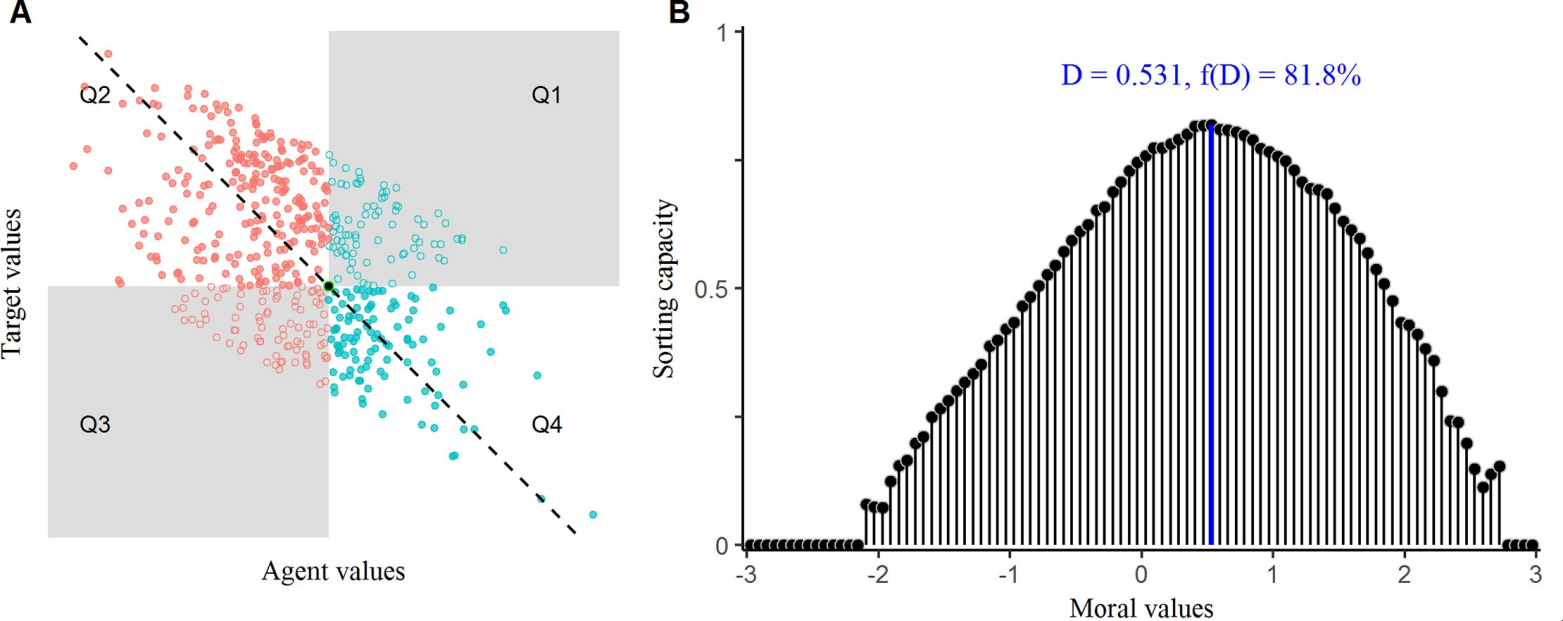
(A) Illustration and (B) results of our optimization method to find the left-right divide. 81.8% of ideological disagreements cross (0.531, 0.531), which we define as the left-right boundary, D.

Ex hypothesi, agent and target values should be negatively correlated as illustrated in Fig 4A, and lie along the disagreement vector, (*agent values—target values*)/2, displayed as a dashed diagonal line. Each position, *p*, on the disagreement vector divides the ideological plane into four quadrants. Quadrant 2 includes the subsample that is more conservative than *p* and views supporters of its rival party as more progressive than *p*. Quadrant 4 contains the subsample that is more progressive than *p* and views supporters of its rival party as more conservative than *p*. In other words, disagreements in Quadrants 2 and 4 “cross” point *p*. Thus, the more disagreements we see in these quadrants for a given point *p*, the more likely that *p* is actually the left-right divide.

Disagreements in the shaded quadrants do *not* cross point *p*. They occur on either side of it. In Quadrant 1 agents and targets are both more progressive than *p*; and similarly, in Quadrant 3, agents and targets are both more conservative than *p*. Therefore, the more disagreements we find in these quadrants, the less likely that *p* is the left-right divide.

This line of reasoning enables us to define the function that our numerical optimization is asked to solve: We sought to find the point along the disagreement vector, {−3,…,3}, that maximizes the relative frequency of disagreements in Quadrants 2 and 4:

$$\sqrt{\frac{Quadrant\;2\times Quadrant\;4}{\left(Quadrant\;1+Quadrant\;2)(Quadrant\;3+Quadrant\;4\right)}}$$

As shown in Fig 4B, we obtained a single optimum: Over four out of five ideological disagreements occurred across the left-right divide, *D* = 0.531.

#### Contrasting the effects of ingroup homophily versus outgroup derogation

We replaced ideological disagreement with agent and target extremity, and reran a model that also included the objectivism and identification measures. The model predicted 24% of the variance in prejudice, *F*(1,1045) = 65.84, *p* < .001 revealing significant effects of both agent (*β* = 0.23 [0.17, 0.28], *t* = 8.02) and target (*β* = 0.15 [0.09, 0.21], *t* = 5.13) extremity on partisan prejudice, *p*s < .001.

Some research has shown that partisan identification serves in part to symbolize one’s ideological commitments. If so, we should expect greater partisan identification among more extreme ideologues, which is indeed what we observed (*β* = 0.12 [0.06, 0.18], *t* = 3.68, *p* < .001; objectivism: *β* = 0.06 [0.00, 0.13], *t* = 1.98, *p* = .048).

This opens up the possibility that the effect of partisan identification on prejudice might be *instrumental*, as some prior research has shown: i.e. partisans are more prejudiced simply as a way of channeling what is ultimately ideological conflict. If partisanship plays this instrumental role, the effects of extremity and objectivism should be primarily indirect via partisan identification.

**Fig 5:**
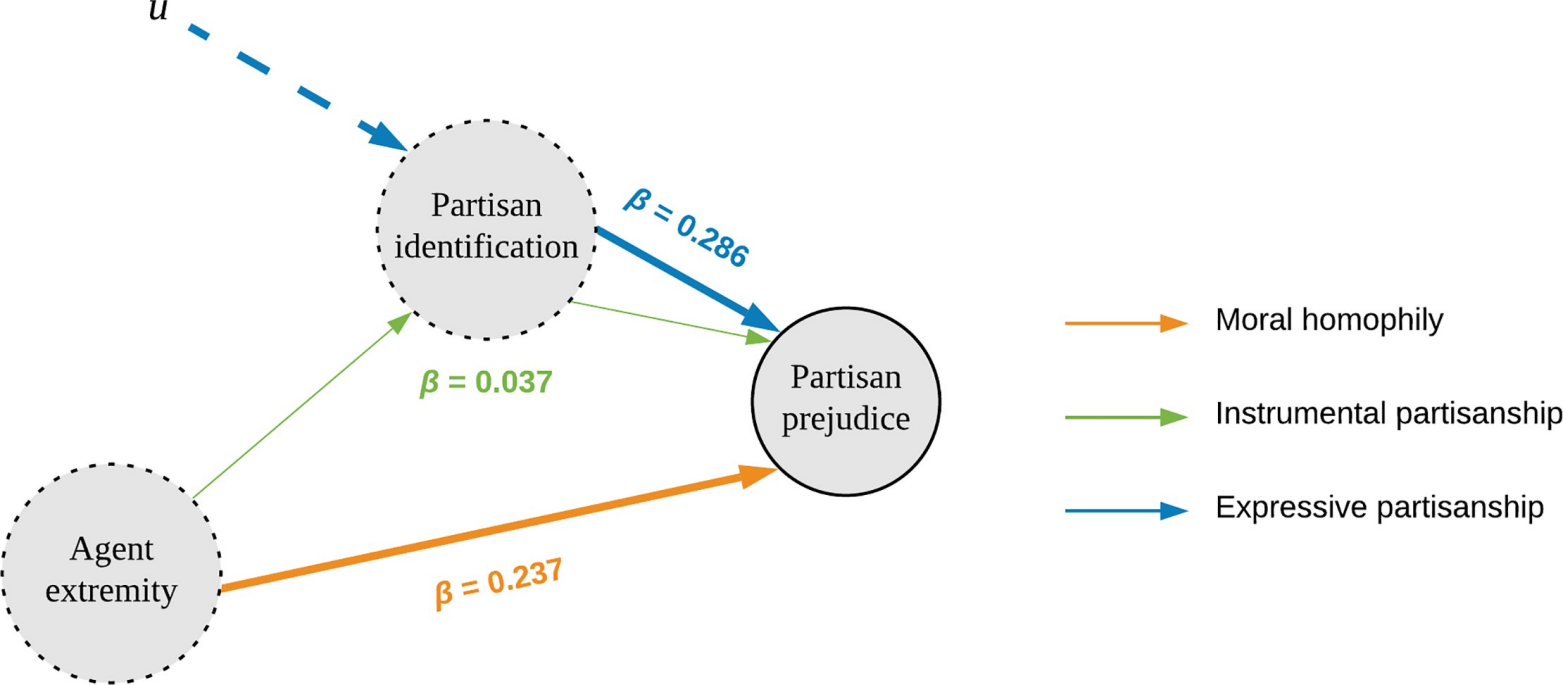
Path model of ideology and identity-based prejudice.

To assess whether partisan identification mediates the observed effects of ideological extremity, we conducted a path analysis using the *lavaan* package (Rosseel, 2012). Collectively, the indirect effect of the morality measures via partisan identification was statistically significant, *β* = 0.088, *z* = 3.13, but accounted for a small fraction of their total effect, *indirect/total* = .177 [.076, .277]. In contrast, the direct effect of the morality measures on partisan prejudice was reasonably large, *β* = 0.416 [0.258, 0.574], *z* = 5.17, as was the independent effect of partisanship, *β* = 0.286 [0.230, 0.337], *z* = 10.38, all *p*s < .001. Taken together, these results suggest that ideological extremity and partisanship play important, but highly distinct, roles in partisan prejudice (see Fig 5).

Looking separately at the morality measures, we found significant direct effects of objectivism, *β* = 0.094 [0.038, 0.150], z = 3.30, target extremity, *β* = 0.140 [0.078, 0.201], *z* = 4.46, and particularly agent extremity, *β* = 0.237 [0.174, 0.300], *z* = 7.37, *p*s < .001. Target objectivism had no effect, *β* = 0.033, *z* = 1.170, *p* = .242. The difference between the direct effects of agent and target extremity was statistically significant, *z* = 4.11, *p* < .001, yielding an answer to our second question. That is, though prejudice depends upon both agent and target extremity, the former effect is significantly stronger. No corresponding difference was found between their indirect effects via identification, *z* = 1.45, *p* = .147.

## Discussion

Past research has given rise to two contrasting theories regarding the emergence of discrimination across party lines. One class of theories posits partisan *identity* as the core contributor to prejudice, while other *issue-based* theories view discrimination as the strategic response to prescriptive disagreement on questions of morality and policy. We conducted a nationally representative survey in order to contrast and extend these models of partisan prejudice.

Our first goal was to deepen our understanding of the issues view, by asking the following question: Are we intolerant toward supporters of rival parties only insofar as we disagree on morality and policy (Hypothesis 1), or does the belief that these disagreements have an objectively right answer exacerbate our prejudice (Hypothesis 2)?

Pre-registered analyses yielded evidence of both associations: Respondents were prejudiced against opposing party targets to the extent that they perceived prescriptive disagreement on a wide range of issues, ranging from gender equality to taxation and immigration policy. Whether respondents thought they were objectively correct also independently predicted prejudice, and both effects were robust to differences in the strength of people’s partisan identities.

Moral philosophers have sometimes speculated that people’s resistance to moral diversity could be the result of a belief in objective moral truth [76], and have even looked to meta-ethical relativism as a promising antidote [49]. Perhaps viewing moral disagreement in a relativist light could help dilute our ingrained prejudice against those with different moral codes. This theory predicts an interactive effect that was entirely absent in our study (Hypothesis 3). Though we replicated past findings that objectivists demonstrate greater prejudice than relativists toward ideologically discordant targets, they were also more prejudiced toward targets with whom they perceived to be in agreement. Thus, at least on the basis of the data reported here, objectivists were simply more intolerant of others overall. It may suggest that efforts to promote tolerance through a common evaluative ground on the partisan divide may clash with folk objectivist tendencies.

Our results dovetail with recent evidence that partisan prejudice is dual in nature (Garrett & Bankert, 2018). Although both partisan identification and ideological disagreement strongly predicted hostility, these effects appeared to be largely independent—a finding that accords with the literature on expressive partisanship ([10], [11]) and also predicts the compounding effect of *sorting* [26], whereby prejudice should be aggravated as ideologues acquire stronger partisan inclinations.

One way of thinking about polarization is that it constitutes mutual and undifferentiated antagonism between polarized ideologues. But, by estimating the location of the left-right divide and leveraging the ideological diversity inherent to a multiparty electorate, we were able to probe further into the nature of partisan prejudice. That is, is prejudice greater *among* or *toward* the opinionated ideologues on either side? Our results pointed first and foremost to agent extremity: Being highly opinionated on moralized prescriptive issues may somewhat inspire fellow citizens’ animosity, but it seems to play an even greater role in making *us* hostile toward them.

We also wish to highlight the principal limitations of our present research. First, the constructs we measured in our study shared substantial variance, raising the question of whether their documented effects on discrimination reflect distinct processes. Alternatively, these effects may stem from broader information-processing biases, such as *meta-cognitive failure* [77] or *overprecision* [71] that could foreseeably yield extremity, objectivism, and an exaggerated sense of disagreement with others (see also [78]).

Second, since our data are strictly cross-sectional, they do not provide direct support for the path model we stipulated. Rather, the assumptions that underlie our path model rest on a larger body of longitudinal and experimental evidence revealing effects of moral conviction on identity [42], [43], [44], and prejudice [16], [24], [25] and of identity on prejudice [11], [12], [26], [15]. Some of these paths are likely to be bidirectional (e.g., partisan identity also shapes supporters’ ideology; see [79]), which raises the question of which specification best synthesizes the overall body of evidence linking morality, identity, and prejudice. However, the purpose of our path analysis was not to advocate, but to assess, a hypothesized mediation path: i.e. we assumed that partisanship *could* play an instrumental role in order to evaluate how large that effect would be. Had we specified the opposite causal path (implying that people’s values are shaped by their partisan identities), our model would already contain the assumption that partisanship plays no instrumental role at all.

Finally, though our study was inspired in part by meta-ethical concepts (e.g., objectivism), we assume that there are other meta-ethical dimensions besides the one we have explored here [80], [81] and recognize that our instrumentalization lacks the nuance with which some philosophers have enriched debates about the nature of morality [82] [83].

In the current climate of political polarization, tempering the undesirable consequences of partisan prejudice should be treated as a social priority. Yet this objective is not easily met without a clearer picture of partisan prejudice, how it arises and how it is sustained. Our present work contributes to this aim by highlighting its dual nature. Partisan prejudice responds to two distinct, though at times co-occurring, motives: one, by which partisans protect their identities through antagonism toward rival parties; and two, by which ideologues tend to withdraw and coalesce in new, more homogeneous moral communities. A deeper understanding of each of these psychological motives could also help to identify effective measures for educators and policy-makers to foster comity and tolerance among the citizenry.

## Supporting information

S1 Appendix Survey methods and materials. S1_Appendix__Survey_Methods_and_Materials.
(PDF)

S2 Appendix Exaggeration of moral differences. S2_Appendix__Exaggeration_of_ moral_differences.
(PDF)

S1 Dataset Rdata data file. 29368ecd-0aa1-49f7-b5eb-d8ff13621931
(RDATA)
