# Supplementary material for: The dual nature of partisan prejudice: Morality and identity in a multiparty system
Source: PLoS One. 2019 Jul 16;14(7):e0219509. doi: 10.1371/journal.pone.0219509 (PMC6634413; doi:10.1371/journal.pone.0219509)
Supplement: S1 Appendix — S1_Appendix__Survey_Methods_and_Materials. (PDF) [file pone.0219509.s001.pdf]

S1 Appendix. Survey methods and materials

Appendix to The Dual Nature of Partisan Prejudice: Morality and Identity in a Multiparty System

Hugo Viciana[*] **Ivar R. Hannikainen** **Antonio Gaitán Torres**

April 19, 2019

1 Introduction

This appendix includes details about previous pilot studies, scales development, reliability of main measures and survey methods for the main study. The project was approved by the Ethics Committee of the Consejo Superior de Investigaciones Cientificas in January 2018. Initially we piloted some of our main ideas for the project in February 2018 on a sample of 165 students of the University Carlos III de Madrid recruited online by email. Further data and materials can be found at the Open Science Framework site for this project (`https://osf.io/kj6ep/`)

2 Previous Facebook ad study

In September 2018, we conducted a previous study, recruiting a convenience sample of 261 Spanish residents (77% women, Mage = 34 years old) through an ad on Facebook. Respondents were asked whether they themselves (Agent condition, n = 137) or supporters of their least-liked party (Target condition, n = 124) agree with each statement. Data and main analyses for the previous online study that we conducted with a convenience sample can be found at the *Open Science Framework* site for this project `https://osf.io/k24g6/`.

To examine the reliability and factor structure of each scale and in order to capture putative moral disagreements between progressive and conservative partisans in Spain, we drafted eighteen statements about controversial moral issues listed in Table 2. In a between subjects design, respondents were asked whether they (n = 137) or supporters of their least-liked party (n = 124) agree with each statement. We conducted a principal components analysis on Self and Target data, and found that two items failed to load onto the principal factor: Surrogacy, and Prostitution.

For our assessment of partisan prejudice, we relied on four distinct social relationships. Respondents in the Facebook ad study were asked how much they would like to establish each type of relationship with an ingroup (i.e., supporters of their preferred party) and an outgroup (i.e., supporters of their disfavored party) member, using separate eight-point scales ranging from 1: "Not at all" to 8: "Very much". Every item loaded onto the principal component, and as a Assortative Sociality measure revealed very good reliability ($\alpha = .83$).

3 Survey methodology

3.1 Participant recruitment

To obtain a representative sample of the Spanish population including sufficient number of supporters of the 4 main political parties, we teamed up with IMOP (https://www.imop.es)–a leading market research and polling firm based in Madrid, Spain.

The survey was conducted with a representative sample of the Spanish adult population with Internet access recruited by IMOP. Panelists were initially recruited via phone through random digit dialing in order to obtain a probabilistic sample of the Spanish population, precluding opt-in voluntary participation. Between October 23rd and November 13th

[*] Shared first authorship Ivar R. Hannikainen & H.V.; Contact: Hugo.Viciana@normalesup.org

S1 Table 1

Item		r	α	PC1	u^2
The voluntary termination of pregnancy is immoral	*	.55	.79	-0.73	0.46
		.71	.94		
Humans should not eat meat; it's unjust.		.46	.80	0.48	0.77
		.59	.95		
Those with the highest earnings should pay proportionately more in taxes.		.46	.80	0.48	0.77
		.59	.95		
Everyone is free to use whichever drugs they choose.		.39	.80	0.80	0.36
		.82	.94		
It is immoral for women to spend more time on household duties than men.		.45	.80	0.64	0.59
		.69	.94		
No immigrant should be deprived of their rights for being 'illegal'.		.61	.79	0.78	0.39
		.81	.94		
Environmental standards should be flexible given the importance of economic growth.	*	.37	.80	-0.69	0.53
		.68	.94		
Burning the Spanish flag is an action that is protected by freedom of expression.		.58	.79	0.68	0.53
		.79	.94		
The right to housing is fundamental, and therefore eviction should be forbidden.		.46	.80	0.48	0.77
		.59	.95		
If a businessperson prefers not to hire Muslim workers, she has the right not to.	*	.63	.79	-0.79	0.37
		.88	.94		
Obedience to the teacher is one of the most important things that children should learn in school.	*	.40	.81	-0.51	0.74
		.59	.95		
Surrogate motherhood should be a right.				-0.10	0.99
Spaniards should be loyal to Spain above other concerns.	*	.65	.79	-0.82	0.33
		.84	.94		
Economic inequality is an evil that must be fought against.		.41	.80	0.75	0.43
		.75	.94		
Bullfighting is of great cruelty and should be eliminated.		.60	.79	0.83	0.31
		.86	.94		
Prostitution is an evil. It must not be regulated. It must be abolished.				0.02	1.00
Despising the Spanish Constitution is obscene.	*	.64	.79	-0.73	0.46
		.75	.94		
There are too many who are receiving unemployment aid and other subsidies and who do not deserve to be given this money.	*	.56	.79	-0.74	0.45
		.79	.94		

S1 table1. List of items for 'moral values' and 'meta-ethical beliefs' task. Statistics for the Facebook ad study. * indicates reverse-scored items. r = item-rest correlation; α = Cronbach's alpha (if removed): PC1 = Principal Component 1 loading; u2 = Uniqueness. r and α are reported separately for the Agent (top) and Target (bottom) condition.

S1 Table 2

Item	reversed	N	M	SD	r	α	PC1	u^2
In-laws - Outgroup		261	3.02	1.54	0.57	0.81	-0.66	0.56
Boss - Outgroup		261	3.00	1.57	0.54	0.82	-0.64	0.59
Teacher - Outgroup		261	2.85	1.55	0.53	0.82	-0.63	0.60
Neighbor - Outgroup		260	3.47	1.36	0.44	0.83	-0.53	0.72
In-laws - Ingroup	*	261	4.50	1.62	0.65	0.80	0.78	0.39
Boss - Ingroup	*	260	4.69	1.61	0.58	0.81	0.71	0.49
Teacher - Ingroup	*	261	4.66	1.64	0.63	0.81	0.76	0.42
Neighbor - Ingroup	*	261	4.01	1.61	0.55	0.82	0.69	0.52

S1 table 2. Partisan prejudice (assortative sociality task). Item statistics for the Facebook ad study. r = item-rest correlation; α = Cronbach's alpha (if removed): PC1 = Principal Component 1 loading; u2 = Uniqueness.

2018, a sample of 1051 Spanish adults (552 women, M. age= 43.8) in the IMOP panel completed the study by visiting a survey website. No more than five reminders were sent to each panelist.

Sampling weights($M = 0.95, SD = 0.25, Min = 0.53, Max = 2.30$) were applied to the final sample to ensure geographical, demographic and socioeconomic representation of the adult Spanish population with Internet access. Three population parameters were employed in computing the frequency weights (via Horvitz-Thomson estimator, inverse probability of selection): distribution by (1) autonomous community(seven geographical regions), (2) sex-and-age, and (3) settlement size(five levels, to represent the distribution of urban and rural voters). A comparison of the demographics of the sample and the official Spanish population statistics can be found in S1 table 3, and S1 table 4.

3.1.1 Data exclusion

Three pre-registered exclusion criteria were implemented by the survey firm prior to delivery to authors: Participants were excluded if they (1) failed to reach the end of the questionnaire, (2) completed the survey in fewer than 200 seconds or failed a simple attention check (to reduce inattentive participation), or (3) reported being ineligible to vote in Spanish elections (to maximize validity).

4 Materials

4.1 Consent form

Bienvenido/a al estudio sobre opiniones y conducta. Este documento describe tus derechos como participante en este estudio online. Te recomendamos que guardes una copia del mismo. Abriendo este enlace en una nueva pestaña, encontrarás una copia pdf de este documento. También puedes imprimir este documento mediante el siguiente botón:

Marco:

Este estudio se enmarca en el ámbito de una investigación científica sobre preferencias sociales dentro del Proyecto Constisuje "La constitución del sujeto en la interacción social: identidad, normas y sentido de la acción desde la perspectiva de la filosofía de la acción, la epistemología y la filosofía experimental", llevado a cabo por investigadores del Consejo Superior de Investigaciones Científicas y la Universidad Carlos III de Madrid y financiado por el Ministerio de Economía y Competitividad (Convocatoria 2015 Programa Estatal de Investigación, Desarrollo e Innovación Orientada a los Retos de la Sociedad).

Procedimiento:

Una vez que entres en el estudio, vamos a pedirte que leas varias afirmaciones (de naturaleza moral, social o sobre preferencias personales) y respondas a unas preguntas sobre ellas. También te haremos una pregunta sobre cómo evalúas a algunos de los principales partidos políticos en España. Para finalizar, vamos a preguntarte algunas cosas sobre ti (variables socioeconómicas habituales en este tipo de estudios como tu género o si eres estudiante) para conocer un poco mejor a los participantes del estudio y poder realizar estadísticas sobre los mismos. Podemos anticiparte que responder a este cuestionario suele llevar entre 5 y 15 minutos.

S1 Table 3

Demographics	Unweighted sample statistics	Spanish population official statistics
Men Ages 18 to 24	4.4%	4.2%
Women Ages 18 to 24	5.7%	4%
Men Ages 25 to 34	8.7%	8%
Women Ages 25 to 34	9.2%	7%
Men Ages 35 to 44	12%	9.8%
Women Ages 35 to 44	12.1%	9.6%
Men Ages 45 to 54	13.8%	9.6%
Women Ages 45 to 54	11%	9.6%
Men Ages 55 to 64	7.4%	7.6%
Women Ages 55 to 64	6.7%	7.9%
Men Ages 65 and older	5.9%	10.1%
Women Ages 65 and older	2.6%	13.3%

S1 Table 4

Demographics	Unweighted sample statistics	Spanish population official statistics
Living in a town <2000 pop.	3.5%	6%
Living in a town pop. 2001-10.000	11.8%	15.1%
Living in a town pop. 10.001-50.000	21.7%	27.1%
Living in a town pop. 50.001-200.000	25.4%	22.5%
Living in a town > 200.000 pop.	37.2%	29.3%

Elegibilidad:

Para participar en este estudio debes tener al menos 18 años. Solo se debe participar una vez.

Posibles riesgos y beneficios:

Aunque es poco frecuente, algunos participantes pueden sentir un leve malestar ante algunas preguntas sobre cuestiones morales o sociales. No obstante, no se ha detectado ningún riesgo ni físico ni psíquico asociado a la participación en este estudio online y aunque la participación en el mismo no te beneficie a ti personalmente, sí esperamos que los resultados que se obtengan hagan avanzar el conocimiento que tenemos sobre ciertos procesos de la conducta social. Esperamos además que la realización de esta encuesta te resulte interesante.

Usos y finalidad de los datos del estudio:

Los datos recabados en este estudio se utilizarán para corroborar hipótesis en el marco de una investigación científica académica para comprender mejor las preferencias sociales. Las estadísticas de los datos podrán formar parte posteriormente de publicaciones científicas (artículos en revistas científicas sujetas a revisión por pares) o divulgativas (artículos divulgativos en publicaciones de acceso libre).

Confidencialidad y anonimidad de los datos:

Las respuestas que facilites serán confidenciales y los datos que se deriven de esas respuestas se usarán únicamente con fines asociados a la investigación académica llevada a cabo por el Consejo Superior de Investigaciones Científicas y universidades asociadas para este estudio (Universidad Carlos III de Madrid; Pontificia UC-Río). Como variable identificatoria indirecta, únicamente se extraerá la dirección IP del equipo desde el que se responde el cuestionario. Dicha información será desvinculada y eliminada posteriormente, anonimizando así completamente las respuestas. Por consiguiente, tus datos serán anonimizados y únicamente formarán parte de las estadísticas que extraeremos del estudio.

Participación voluntaria:

La participación en este estudio es completamente voluntaria. Eres libre para rechazar participar en este estudio online, así como para abandonarlo en cualquier momento y por cualquier motivo sin que eso suponga ninguna penalización ni la pérdida de ninguno de los derechos que se recogen en esta hoja de información. Para abandonar el estudio basta con interrumpir la navegación online y cerrar la encuesta. Si tras finalizar el estudio, deseas revocar tu acuerdo de participación, puedes hacerlo escribiendo a la dirección de contacto aquí abajo.

Dudas, preguntas y dirección de contacto:

Para cualquier duda, queja, pregunta o sugerencia sobre este estudio puede ponerse en contacto con el investigador principal del estudio Hugo Viciana (IESA-CSIC) en la dirección Hviciana@iesa.csic.es. También puede ponerse en contacto con el director y coordinador del proyecto Antonio Gaitán (Universidad Carlos III de Madrid), en la dirección agaitan@hum.uc3m.es

Acuerdo de participación:

Al cliquear el botón de abajo estás indicando que has leído el consentimiento informado que te presentamos aquí arriba, que tienes al menos 18 años y que acuerdas participar libremente en este estudio.

¿Eres mayor de 18 años y aceptas participar en este estudio?

- Sí (1)
- No (2)

4.2 Identification task

En la actualidad, hay cuatro agrupaciones políticas principales en España: PP, PSOE, Unidos Podemos y Ciudadanos. Ordénalas según el grado en que te identificas con cada partido (de mayor a menor).

______ PP (1)

______ PSOE (2)

______ Unidos Podemos (3)

______ Ciudadanos (4)

There are currently four main political parties in Spain: PP, PSOE, Unidos Podemos y Ciudadanos. Re-order them according to the degree to which you identify with each party (from most to least).

______ PP (1)

______ PSOE (2)

______ Unidos Podemos (3)

______ Ciudadanos (4)

En la actualidad, hay cuatro agrupaciones políticas principales en España: PP, PSOE, Unidos Podemos y Ciudadanos. ¿En qué grado te identificas con cada partido? (Por favor, responde en una escala de 0 a 100)

Currently, there are four main political parties in Spain: PP, PSOE, Unidos Podemos and Ciudadanos. To what extent do you identify with each of these parties (Please answer on a scale from 0 ('Not at all') to 100 ('Absolutely'))

4.3 Moral Values section

4.3.1 Self-report Agent moral views:

Ahora, según tus valores y tu opinión propia, en qué medida estás personalmente de acuerdo o en desacuerdo con cada una de las siguientes afirmaciones.

Now, according to your values and personal opinions, to what extent do you agree or disagree with each of the following statements.

See Item List.

4.3.2 Self-report Agent meta-ethical beliefs:

Para cada afirmación, imagina que dos personas discuten sobre si es verdadera o falsa. Una de ellas piensa que es verdadera y la otra piensa que es falsa. En tu opinión, ¿pueden ambos tener razón o uno de ellos se equivoca?

For each statement, imagine that two people are arguing about whether the statement is true or false. One person thinks it is true and the other thinks it is false. In your opinion, can both be right or is one of them mistaken?

See Item List.

4.3.3 Target representation moral views:

Piensa en las personas que, de entre los cuatro partidos principales, simpatizan más con [least-liked party]. En esta sección, queremos saber si eres capaz de averiguar qué suelen responder los que simpatizan con [least-liked party].

¿En qué medida suelen estar de acuerdo o en desacuerdo con cada una de las siguientes afirmaciones las personas que simpatizan con [least-liked party]? Responde como piensas que respondería normalmente alguien que simpatiza con [least-liked party]

***

Think about people who, from among the four main political parties, are most in favor of [least-liked party]. In this section, we want to know whether you can guess what people who support [least-liked party] tend to believe.

To what extent do people who support [least-liked party] agree or disagree with each of the following statements? Answer the way you think someone who supports [least-liked party] would normally answer.

See Item List.

4.3.4 Target representation meta-ethical beliefs:

Piensa en las personas que simpatizan más con [least-liked party]. En esta sección, queremos saber si eres capaz de averiguar qué actitudes suele tener alguien que simpatiza con [least-liked party].

Para cada afirmación, imagina que dos personas discuten sobre si es verdadera o falsa. Una de ellas piensa que es verdadera y la otra persona piensa que es falsa. ¿Qué piensas que opinaría una persona que simpatiza con [least-liked party] sobre ese desacuerdo? Para alguien que simpatiza con [least-liked party], ¿pueden ambos tener razón o uno de ellos se equivoca? Responde como crees que suele responder alguien que simpatiza con [least-liked party].

***

Think about people who, from among the four main political parties, are most in favor of [least-liked party]. In this section, we want to know whether you can guess what people who support [least-liked party] tend to believe.

For each statement, imagine that two people are arguing about whether the statement is true or false. One person thinks it is true and the other thinks it is false. What would someone who supports [least-liked party] think about their disagreement? For someone who supports [least-liked party], can both be right or is one of them mistaken? Answer the way you think someone who supports [least-liked party] would normally answer.

See Item List.

4.4 Item List:

1. “Interrumpir voluntariamente el embarazo es inmoral.”
2. “El ser humano no debería comer carne; es injusto.”
3. “Los que más ingresan deben contribuir proporcionalmente más en impuestos.”
4. “Cada uno es libre de usar las drogas que quiera.”
5. “Es intolerable que la mujer dedique más horas a tareas domésticas.”

6. “Ningún inmigrante debería ser privado de derechos por ser ‘ilegal’.”
7. “La criterios medioambientales deberían ser flexibles ante la importancia del crecimiento económico.”
8. “Quemar la bandera de España es una acción protegida por la libertad de expresión.”
9. “El derecho a la vivienda es básico y, por tanto, el desahucio debería estar prohibido.”
10. “Si un empresario no quiere contratar a trabajadores musulmanes, está en su derecho.”
11. “La obediencia al maestro es de lo más importante que los niños deberían aprender en la escuela”
12. "La maternidad subrogada es decir, que una mujer se embarace y dé a luz al hijo de otra pareja debería ser un derecho."
13. “El té está más rico que el café.”
14. “Está mal causar sufrimiento injustificado y gratuito a personas inocentes”.
15. “La tierra en realidad es plana y el sol gira alrededor de ella.”
16. “Los españoles deberían ser leales a España por encima de otras consideraciones.”
17. “La desigualdad económica es un mal contra el que se debe luchar.”
18. “La tauromaquia es de una gran crueldad y debería eliminarse”
19. “La prostitución es un mal y como tal no se regula, se debe abolir.”
20. “Despreciar la Constitución española es indecente”
21. “Hay demasiados que están cobrando el paro y otras ayudas y que no se merecen que les den este dinero”

***

1. *“The voluntary termination of pregnancy is immoral.”*
2. *“Humans should not eat meat; it’s unjust.”*
3. *“Those with the highest earnings should pay proportionately more in taxes.”*
4. *“Everyone is free to use whichever drugs they choose.”*
5. *“It is immoral for women to spend more time on household duties than men.”*
6. *“No immigrant should be deprived of their rights for being ‘illegal’.”*
7. *“Environmental standards should be flexible given the importance of economic growth.”*
8. *“Burning the Spanish flag is an action that is protected by freedom of expression.”*
9. *“The right to housing is fundamental, and therefore eviction should be forbidden.”*
10. *“If a businessperson prefers not to hire Muslim workers, she has the right not to.”*
11. *“Obedience to the teacher is one of the most important things that children should learn in school”*
12. *"Surrogate motherhood, that is, that a woman gets pregnant and gives birth to the child of another couple should be a right."*
13. *“Tea tastes better than coffee.”*
14. *“It is wrong to cause unjustified and gratuitous suffering to innocent people.”*
15. *“The Earth is flat and the sun gravitates around the Earth.”*
16. *“Spaniards should be loyal to Spain above other concerns.”*
17. *“Economic inequality is an evil that must be fought against.”*
18. *“Bullfighting is of great cruelty and should be eliminated”*
19. *“Prostitution is an evil and as such it must not be regulated. It must be abolished.”*
20. *“Despising the Spanish Constitution is obscene”*
21. *“There are too many who are receiving unemployment aid and other subsidies and who do not deserve to be given this money”*

4.5 Partisan Prejudice. Assortative Sociality task

Del 1 (me disgustaría mucho) al 8 (me gustaría mucho), ¿cuánto te gustaría o te disgustaría...

Que un hermano/a o hijo/a tuya se casase con un simpatizante del [least-liked party]?

Tener un jefe o jefa que fuera simpatizante del [least-liked party]?

Que un simpatizante del [least-liked party] fuese profesor de tus hijos en primaria?

Que un simpatizante del [least-liked party] fuese tu médico?

Que tu vecino más cercano fuese un simpatizante del [least-liked party]?

***

From 1 (I would dislike it very much) to 8 (I would dislike it very much), ¿how would you like it:.

For our brother/sister or son/daughter to marry someone who supports [least-liked party]?

For your boss at work to support [least-liked party]?

For your children's school teacher to support [least-liked party]?

For your doctor to support [least-liked party]?

For your next-door neighbor to support [least-liked party]?

4.5.1 Attribution of moral badness:

En tu opinión, ¿cómo de acuerdo o desacuerdo estás con la afirmación siguiente?

"Alguien que simpatice con [least-liked party] es una mala persona"

In your opinion, to what extent do you agree or disagree with the following statement?

"Someone who supports [least-liked party] is a bad person?"

4.5.2 Demographic information:

1. ¿Podrías, por favor, indicarnos tu género?

1. *Please indicate your gender.*

2. ¿Cuántos años cumplió Ud. en su último cumpleaños?

2. *How old are you (in years)?*

3. ¿Has nacido en España o tienes nacionalidad española o eres residente y puedes votar aquí?

3. *Were you born in Spain, or have Spanish nationality, or are a resident who can legally vote in Spain?*

4. ¿Con qué frecuencia asistes a misa u otros oficios religiosos (sin contar las ocasiones relacionadas con ceremonias de tipo social, tipo bodas, funerales, comuniones)?

4. *How often do you go to mass or attend other religious services (setting aside social ceremonies, such as weddings, funerals or communions)?*

5. Si estás leyendo esta pregunta, responde 4:

5. *If you are reading this, please select the answer 4:*

6. ¿De cuántos ingresos has dispuesto en el último mes?

6. *What was your income last month?*

7. ¿Qué estudios o formación has podido completar?

7. *What is your educational attainment?*

8. ¿Hay algo que te gustaría decirnos sobre qué te ha parecido este estudio?

8. *Is there anything you would like to tell us about your experience in this study?*
