# Supplementary material for: The dual nature of partisan prejudice: Morality and identity in a multiparty system
Source: PLoS One. 2019 Jul 16;14(7):e0219509. doi: 10.1371/journal.pone.0219509 (PMC6634413; doi:10.1371/journal.pone.0219509)
Supplement: S2 Appendix — S2_Appendix__Exaggeration_of_ moral_differences. (PDF) [file pone.0219509.s002.pdf]

S2 APPENDIX. EXAGGERATION OF MORAL DIFFERENCES

APPENDIX TO THE DUAL NATURE OF PARTISAN PREJUDICE: MORALITY AND IDENTITY IN A MULTIPARTY SYSTEM

Hugo Viciana* **Ivar R. Hannikainen** **Antonio Gaitán Torres**

April 19, 2019

1 Introduction

Is polarization partly a matter of how we perceive others? Some studies seem to suggest something like that might be going on (Fiorina et al., 2008), even in the domain of morality and moral differences (Graham et al., 2012). According to this thesis, real moral differences would not be as important as perceived moral differences. In turn, misrepresented moral differences could be the basis of a whole series of dysfunctions in public debates and civic life. If moral and meta-ethical differences can promote intolerance, studying more systematically the misrepresentation of these differences becomes a priority. At the origin of these misrepresentations there may be different operating processes that should be better known. In what follows, we present some results of exploratory studies in relation to our study of the moral roots of partisan prejudice.

2 Do people misrepresent their ideological rivals?

In line with previous research(Graham et al., 2012), targets appeared to be misrepresented when compared to their self-reports. Target extremity vastly exceeded agent extremity in a paired t-test, $t(1050) = 29.7, p < .001, dz = 0.92$, and separate analyses by political party revealed that the discrepancy was larger for conservative than progressive targets: In particular, right-leaning participants appeared to perceive Unidos Podemos supporters as more progressive than the latter self-reported, $t(751.7) = -2.63, p = .009, d = -0.19$. In turn, left-leaning participants perceived both PP, t(342.2) = 14.97, $p < .001, d = 1.11$, and Ciudadanos, $t(339.93) = 18.30, p < .001, d = 1.34$, supporters as much more conservative than the latter self-reported–with differences exceeding one standard deviation.

We observed a corresponding discrepancy on our measure of metaethical beliefs: Respondents viewed targets as more objectivist than themselves, paired $t(1050) = 17.55, p < .001, dz = 0.54$. Right and center-right voters were viewed as much more objectivist than they reported, ($d_{PP} = 0.81, d_{Ciudadanos} = 0.86$), while the corresponding effect for left voters was moderate in size ($d_{UnidosPodemos} = 0.31$).

3 Misrepresentation, moral objectivism and partisan prejudice

Participants' agent-objectivism correlates positively with their distortion scores, meaning that those participants which gave more intuitive objectivist responses to the sociomoral issues also tend to misrepresent more the political outgroup moral attitudes ($r = .15, 95\% CI[.09, .21], p < .001$), which suggests a possible "false dissensus effect[2]" when characterizing the outgroup, $r = .15, 95\% CI[.09, .21], p < .001$). After checking through linear regression models, this positive relationship held after controlling for several possible confounding factors (support for different political groups, agent-extremity, and degree of partisan identification).

We also observed by-issue differences in distortion. Distortion was greater on core issues (on which participants held objectivist beliefs; see Supplementary Table below).

*Shared first authorship Ivar R. Hannikainen & H.V.; Contact: Hugo.Viciana@normalesup.org

[2]Analogous to the 'false consensus effect' (Marx & Miller, 1987) shown in research regarding 'naive realism'.

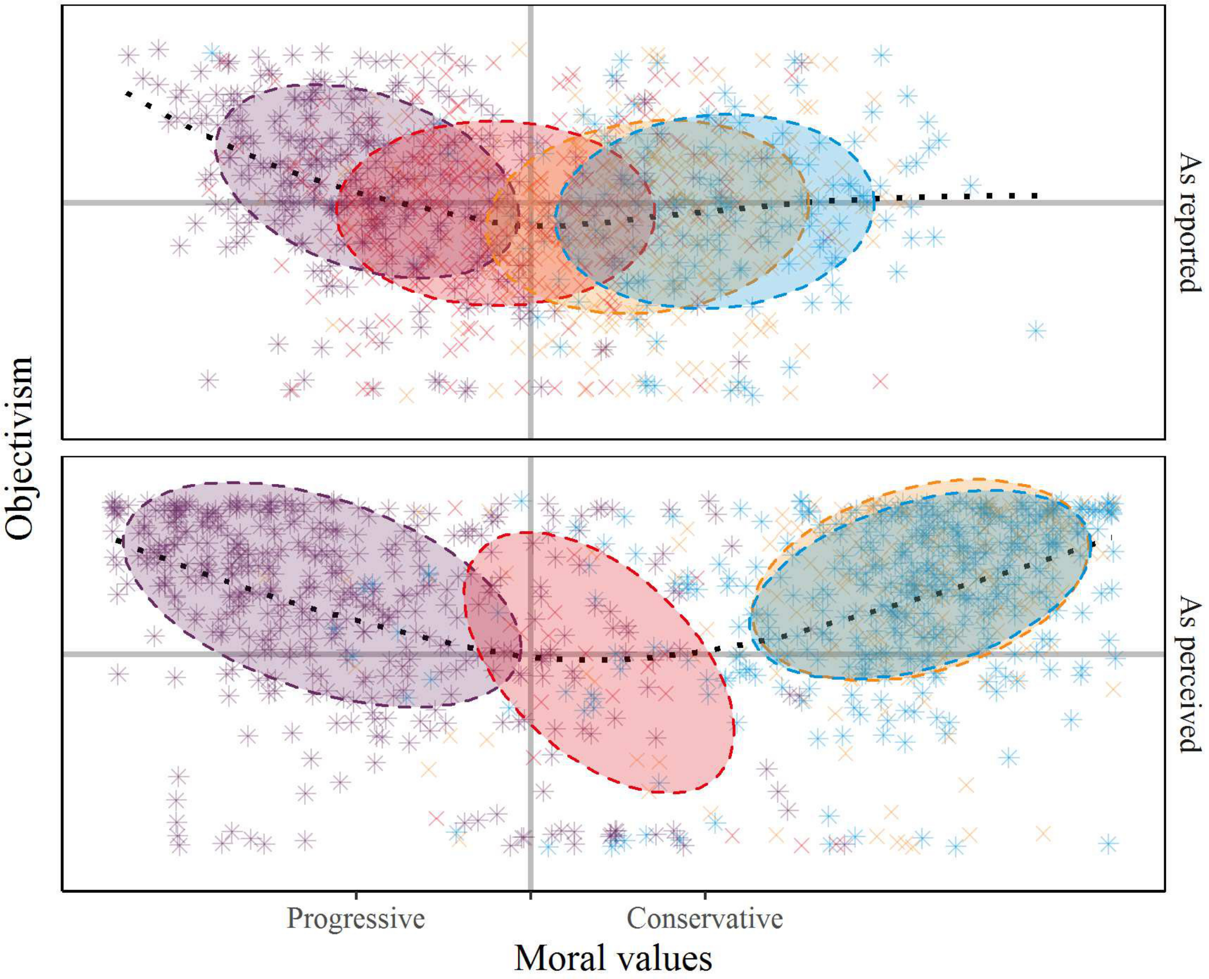

Figure 1: Objectivism and moral values by political parties. The upper plot displays self-reported views by preferred party, while the lower plot displays target representations by disfavored party. Solid lines represent population means, and axis ticks reflect a 1SD shift, in the Self condition. A LOESS regression line describes the relationship between moral values and objectivism in each condition. Shaded ellipses encircle the 68% CI (standard deviation) of the party means.

Why self-reports and rival assessments of moral values vary so markedly remains a puzzle for further work to unravel. In line with previous research, we found a left-right asymmetry: Conservatives were perceived as much more reactionary than they reported, while liberals were only perceived as somewhat more progressive than they reported being. However, we must also note the possibility that the observed discrepancies reflect systematic inaccuracies in self-report–for instance, if reactivity leads conservative participants to downplay their moral dissent and conviction. It could be that either 1) a 'misunderstanding account' where misperceptions about others' moral positions and moral motives fuel political hostility is the best explanation; or 2) a 'motivational account' where a motivation to advance one's political agenda also leads to strategically deforming and caricaturing others' moral positions is driving the observed effect; or 3) that precisely the fear of rejection and discrimination by others leads, at least some people, to publicly moderate their moral positions, partly hiding their private preferences. This latter 'social desirability' account would suggest that what we call distortion – the difference between attributed and self-reported moral judgments – might not be as high as registered by our way of measuring it, since at least some participants would be moderating their moral positions in response to social pressure. Additionally it could be that the 'misunderstanding account' is on the right track and that supporters of different parties do actually have different levels of accuracy and precision in their perception of political competitors' moral positions. Some of the sorting dynamics referred in previous research could supplement this account (Lang & Pearson-Merkowitz, 2015).

S2 Table 1

	UP	PSOE	Cs	PP
Mean Distortion Target.	2.08 (.04)	1.86 (.06)	1.55 (.06)	1.62 (.1)
Core issue UP & PSOE ("Economic inequality is an evil which must be fought against").	3.15↑ (.08) 86%	2.55↑ (.1) 77%	0.98↓ (.07) 66%	1.05↓ (.1) 57%
Core issue UP & PSOE ("It is immoral for a woman to spend more time on house chores")	2.31↑ (.07) 72%	1.89↑ (.07) 69%	1.44↓ (.07) 48%	1.61↓ (.11) 47%
Core issue Cs & PP ("Despising the Spanish Constitution is obscene")	1.44↓ (.04) 51%	1.46↓ (.06) 44%	1.59↑ (.06) 68%	1.82↑ (.1) 69%
Core issue Cs & PP ("Burning the Spanish flag is protected by freedom of speech")	1.61↓ (.07) 61%	1.59↓ (.08) 48%	1.95↑ (.07) 67%	2.05↑ (.08) 78%

Supplementary Table 1 S2. Distortion agent-target for moral judgments. Mean discrepancy for supporters of each political group across all issues on the first row. Several examples of distortion values for specific core sociomoral issues are shown below. Standard errors are shown in parentheses. Percentages shown represent percent of respondents who identify with each political group and who gave the intuitive objectivist answer for the issue in question (agent-objectivism). Arrows indicate whether value is below average or above average for that group.

4 Do we observe left-right symmetry or asymmetry in partisan prejudice?

To evaluate whether progressives and conservatives differ in partisan prejudice, we report two analyses employing participants' moral values (i.e., conservative values < 0 < progressive values) and unfolded moral disagreement (preserving its sign: i.e., positive values = target more conservative than agent; negative values = target more progressive than agent).

In each model below, we enter both linear and quadratic terms. The quadratic term will describe any U-shaped differences–between centrists and ideologues on either side. The linear term will capture any left-right slope after accounting for any quadratic curvature. Thus, the quadratic term can be said to detect symmetric effects, while the linear term can be thought of as capturing asymmetric left-right differences.

First, we regress prejudice on unfolded-disagreement, unfolded-disagreement^2, and both measures of objectivism. This model revealed a quadratic effect of disagreement, $t = 3.69, p < .001$, but no linear effect, $t = 0.03, p = .98$. (The model also replicated the effect of agent objectivism, $B = 0.08, t = 4.43, p < .001$, and the non-significant effect of target objectivism, $B = 0.01, t = 0.70, p = .48$.)

Our second analysis of asymmetry in partisan prejudice involved the sixteen-item agent and target means of moral values. We regressed partisan prejudice on a total ten predictors, five each from the agent and target blocks: (1) objectivism, (2) linear and (3) quadratic moral values, and (4-5) both interactions between objectivism and moral values. The model yielded the following results:

Thus, re-analyzing the data without folding moral values indices at the ideological center largely replicated our results: We found highly significant quadratic effects of agent and moral values (ps < .001), but no linear effects (ps = .68, .28).

References

[1] Fiorina, Morris P., Samuel A. Abrams, and Jeremy C. Pope. Polarization in the American public: Misconceptions and misreadings. *The Journal of Politics*, 2008, 70(2), 556-560.

[2] Graham, Jesse, Brian A. Nosek, and Jonathan Haidt. The moral stereotypes of liberals and conservatives: Exaggeration of differences across the political spectrum. *PloS One* 7.12 (2012): e50092.

S2 Table 2

	Regression term	Coefficient	t-value	p-value
Agent	Objectivism	0.07**	3.82	.000
	Moral Values	-2.51	-0.41	.68
	Moral Values^2	27.83**	4.92	.000
	Objectivism × Moral Values	0.38	0.67	.50
	Objectivism × Moral Values^2	-1.01	-1.88	.06
Target	Objectivism	0.04	1.68	.093
	Moral Values	-7.88	-1.09	.28
	Moral Values^2	13.43*	2.17	.03
	Objectivism × Moral Values	0.23	0.42	.67
	Objectivism × Moral Values^2	-0.41	-0.86	.39

Supplementary table 2 S2. Unfolded linear and quadratic terms. See text for explanation.

[3] Lang, Corey, and Shanna Pearson-Merkowitz. Partisan sorting in the United States, 1972–2012: new evidence from a dynamic analysis. *Political Geography* 48 (2015): 119-129.
